# High-resolution melting analysis of the common c.1905+1G>A mutation causing dihydropyrimidine dehydrogenase deficiency and lethal 5-fluorouracil toxicity

**DOI:** 10.3389/fgene.2012.00312

**Published:** 2013-01-17

**Authors:** Emma Borràs, Emma Dotor, Àngels Arcusa, Maria J. Gamundi, Imma Hernan, Miguel de Sousa Dias, Begoña Mañé, José A. G. Agúndez, Miguel Blanca, Miguel Carballo

**Affiliations:** ^1^Molecular Genetics Unit, Hospital de TerrassaTerrassa, Spain; ^2^Oncology Service, Hospital de TerrassaTerrassa, Spain; ^3^Department of Pharmacology, Faculty of Veterinary, Universidad de ExtremaduraCáceres, Spain; ^4^Allergy Service, Hospital Carlos HayaMálaga, Spain

**Keywords:** dihydropyrimidine dehydrogenase, DPD, *DPYD*, 5-fluorouracil, 5-FU, capecitabine, toxicity, HRM

## Abstract

Dihydropyrimidine dehydrogenase (DPD) deficiency is a pharmacogenetic syndrome associated with life-threatening toxicity following exposure to the fluoropyrimidine drugs 5-fluorouracil (5-FU) and capecitabine (CAP), widely used for the treatment of colorectal cancer and other solid tumors. The most prominent loss-of-function allele of the *DPYD* gene is the splice-site mutation c.1905+1G>A. In this study we report the case of a 73-year old woman with metastatic colorectal cancer who died from drug-induced toxicity after the first cycle of 5-FU-containing chemotherapy. Her symptoms included severe neutropenia, thrombocytopenia, mucositis and diarrhea; she died 16 days later despite intensive care measures. Post-mortem genetic analysis revealed that the patient was homozygous for the c.1905+1G>A deleterious allele and several family members consented to being screened for this mutation. This is the first report in Spain of a case of 5-FU-induced lethal toxicity associated with a genetic defect that results in the complete loss of the DPD enzyme. Although the frequency of c.1905+1G>A carriers in the white population ranges between 1 and 2%, the few data available for the Spanish population and the severity of this case prompted us to design a genotyping procedure to prevent future toxic effects of 5-FU/CAP. Since our group had previously developed a high-resolution melting (HRM) assay for the simultaneous detection of *KRAS*, *BRAF*, and/or *EGFR* somatic mutations in colorectal and lung cancer patients considered for EGFR-targeted therapies, we included the *DPYD* c.1905+1G>A mutation in the screening test that we describe herein. HRM provides a rapid, sensitive, and inexpensive method that can be easily implemented in diagnostic settings for the routine pre-therapeutic testing of a gene mutation panel with implications in the pharmacologic treatment.

## Introduction

Dihydropyrimidine dehydrogenase (DPD; EC 1.3.1.2) is the initial rate-limiting step in the catabolism of endogenous pyrimidines, as well as in fluoropyrimidine drugs such as 5-fluorouracil (5-FU) and its oral prodrug capecitabine (CAP), widely used in the treatment of colorectal cancer and other solid tumors. With a predominant expression in the liver, DPD rapidly catalyzes the reduction of more than 80% of the 5-FU administered (Heggie et al., 1987); thus, a reduced enzymatic activity increases the half-life of the drug, resulting in excess accumulation and toxicity (Ezzeldin and Diasio, 2004; Lee et al., 2004; van Kuilenburg et al., 2008). DPD activity is highly variable in the population, as it depends on many factors such as gender, circadian rhythms, drug interactions and genetic polymorphisms (Mercier and Ciccolini, 2006); with an estimated 3–5% of individuals experiencing low or deficient activity (Yen and McLeod, 2007).

DPD deficiency (OMIM 274270) is an autosomal recessive disorder described in pediatric patients presenting with thymine-uraciluria and major symptoms of convulsion and psychomotor retardation, although asymptomatic cases also exist (Webster et al., 2001). A common trait in these patients is the complete deficiency of DPD due to homozygosity or compound heterozygosity of inactivating mutations in the *DPYD* gene (van Kuilenburg et al., 1999). Likewise, the complete or partial loss of DPD function in cancer patients carrying *DPYD* mutant alleles is known to cause severe life-threatening hematologic and gastrointestinal toxicity after 5-FU administration (Amstutz et al., 2011). Accounting for 50–75% of severe 5-FU-related toxicities (Ciccolini et al., 2010), DPD deficiency has been defined as a pharmacogenetic syndrome and is on the FDA's list of approved biomarkers (http://www.fda.gov/Drugs/ScienceResearch/ResearchAreas/Pharmacogenetics/ucm083378.htm). Lethal toxicities have been reported in DPD deficient patients treated with either 5-FU (Milano et al., 1999; Raida et al., 2001; van Kuilenburg et al., 2001, 2003; Ezzeldin et al., 2003; Magné et al., 2007; Morel et al., 2007; Saif et al., 2007a; Gross et al., 2008) or CAP (Ciccolini et al., 2006; Largillier et al., 2006; Saif et al., 2007b; Deenen et al., 2011). Indeed, combined pharmacogenetic syndromes with a fatal outcome have been associated with concomitant mutations in the *DPYD* and *UTG1A1* genes (Steiner et al., 2005; Mounier-Boutoille et al., 2010).

*DPYD* is a 843-kb, single copy gene located on chromosome 1p22 that comprises 23 exons and appears to be highly polymorphic, with more than 50 variants reported (Takai et al., 1994; Yokota et al., 1994; Wei et al., 1998). However, only three individual variants have been consistently associated with 5-FU toxicity in case-control studies (Amstutz et al., 2011): the two nonsynonymous substitutions c.1679T>G (I560S) and c.2846A>T (D949V), which result in low enzyme activity but are very rare; and c.1905+1G>A (formerly IVS14+1G>A or DPYD^*^2A), a point mutation in the splice donor site that results in a 165-bp deletion in the mRNA, due to skipping of exon 14, and lack of functional DPD expression (Meinsma et al., 1995; Vreken et al., 1996; Wei et al., 1996).

The c.1905+1G>A mutation has been the most frequently studied in the context of 5-FU toxicity as it proved to be the most prevalent among patients with complete DPD deficiency (52%) (van Kuilenburg et al., 1999) and was detected in 24% of cancer patients suffering grade 4 leucopenia, with the majority of them being heterozygous (Raida et al., 2001). Moreover, large general population screenings for the c.1905+1G>A mutation showed 1–2% of heterozygous carriers (Raida et al., 2001; van Kuilenburg et al., 2001), rendering this allele attractive for routine mutation screening. However, subsequent studies indicated a north-south gradient in Europe, so the proportion of 5-FU toxicity cases explained by the c.1905+1G>A variant varied greatly due to population frequency differences and sampling effects (Amstutz et al., 2011).

In Spain, the only studies addressing the prevalence of this mutation were conducted in colorectal cancer patients treated with 5-FU (Paré et al., 2010) and CAP (Salgado et al., 2007), showing heterozygote frequencies of 0% (0/234) and 1.7% (1/58), respectively. Some cases of 5-FU/CAP-induced severe toxicity in DPD-deficient patients have been reported in our country, mainly via communications at congresses or in pharmacy journals (Gironés Sarrió et al., 2005; López Sobella et al., 2008; Rubio Salvador et al., 2012). In the most recent report, and the only study to perform a genetic analysis, one toxic death was attributed to heterozygosity of an unspecified mutant *DPYD* allele (Rubio Salvador et al., 2012). It is also worth mentioning the case of a Spanish woman, reported in a French study, who died from 5-FU toxicity due to heterozygosity for the c.464T>A mutation (Morel et al., 2007).

The relatively high frequency of the c.1905+1G>A variant, with 1.3% of heterozygote carriers according to 1000 Genomes data (rs3918290 polymorphism in 1000GENOMES:EUR population at http://browser.1000genomes.org), together with the widespread use of 5-FU/CAP and the severity of the associated toxicities, prompted us to develop a fast and reliable method to identify high-risk individuals prior to undergoing pyrimidine-based chemotherapy. Since we had previously developed a high-resolution melting (HRM) assay to detect somatic *KRAS*, *BRAF*, and *EGFR* mutations in tumor samples from patients considered for EGFR-targeted therapies (Borràs et al., 2011), we included the detection of the *DPYD* c.1905+1G>A mutation in this screening test.

The HRM method is based on a PCR amplification using a saturating intercalating dye, followed by DNA strand separation in a temperature gradient, during which the fluorescence is registered with a high resolution. Thus, the melting curves obtained for homozygous and heterozygous samples differ significantly. Likewise, for somatic mutations, the presence of mutated alleles results in abnormal melting profiles.

Herein, we report the first case in Spain of a patient with 5-FU-induced lethal toxicity due to homozygosity for the c.1905+1G>A mutation, and describe a HRM assay for the routine testing of cancer patients prior to 5-FU/CAP therapy.

## Materials and methods

### DNA samples

The study was conducted in accordance with the Declaration of Helsinki and was approved by the internal Clinical Research Ethics Committee (CEIC) of the Hospital de Terrassa (Spain). Informed consent was obtained from all the participants and has been archived by the authors. Genomic DNA samples were obtained from a patient who died from 5-FU-induced toxicity and her close relatives. In the case of the index patient, the DNA was extracted from stored frozen blood samples and the consent for genotyping was provided by the relatives after the patient's death. DNA isolation from peripheral blood lymphocytes was performed automatically by the MagNaPure Compact Instrument (Roche Applied Science, Barcelona, Spain) according to the manufacturer's protocol.

### PCR amplification and sequencing

A newly designed forward primer (5′-TATGGCCCTGGACAAAGCTC-3′) was combined with an existing reverse primer for *DPYD* exon 14 (5′-CAGCAAAGCAACTGGCAGATT-3′) (Kumar et al., 2007) to generate a 239-bp amplicon. Primer specificity and melting temperatures were analyzed using Primer-BLAST software (http://www.ncbi.nlm.nih.gov/tools/primer-blast). PCR amplification was conducted in a 50 μl final volume containing: 1x PCR buffer, 1.5 mM MgCl_2_, 500 nM primers, 2 μl genomic DNA (32 ng to 1.7 μg), 200 μM dNTPs, 2.5 U of BioTaq polymerase (Bioline, Ecogen, Barcelona, Spain), and PCR grade water. The program conditions were: 5 min at 95°C followed by 35 cycles of 30 s at 95°C, 30 s at 56.6°C and 1 min at 72°C. PCR products were analyzed by 1.5% agarose gel electrophoresis, column purified with the High Pure PCR Product Purification Kit (Roche) and submitted to StabVida (Oeiras, Portugal) for direct sequencing on a 3730XL ABI DNA sequencer (Applied Biosystems, Foster City, CA) using the Big Dye terminator V1.1 DNA sequencing kit.

### Design of HRM primers

First, the primers used for PCR amplification were tested to ensure good genotype discrimination in a LightCycler® 480 platform (Roche) using the previously described HRM assay (Borràs et al., 2011). After optimization of the touchdown PCR annealing temperature range, the DNA samples were successfully amplified and heterozygous carriers of the c.1905+1G>A mutation could be easily identified. However, the melting profiles of mutant and wild-type homozygous samples were almost identical. Alternative primers were designed in order to obtain shorter amplicons, in which a nucleotide change would have a greater effect on the curve shape, with a single melting domain and a low level of secondary structure, according to Stitchprofiles.uio.no (http://stitchprofiles.uio.no) and DINAMelt Web Server (http://mfold.rna.albany.edu/?q=DINAMelt) predictions, but the initial results could not be improved. We therefore decided to use the above mentioned primers but to spike all samples with a known amount of wild-type DNA from a control individual to ensure differentiation of homozygous variants, as suggested in LightCycler® 480 Technical Note No. 1 (2008).

### HRM assay

Samples were diluted at the same concentration and spiked with 0.5 volumes of wild-type DNA, so the mutant:wild-type allele ratio (2:1 in mutant homozygous, 1:2 in heterozygous, and 0:3 in wild-type samples) maximized the ability to discriminate genotypes. Test samples were assayed in triplicate using the LightCycler® 480 system, and negative (non-template) and wild-type controls were included in each experiment. Each 10-μl reaction contained about 30 ng DNA diluted in 1.8 μl, 1x HRM mix (Roche), 3 mM MgCl2, and 200 nM HPLC-purified primers. Touchdown PCR and melting conditions were: 95°C for 10 min; 45 cycles of 95°C for 10 s, 60-53°C (1°C/cycle) for 15 s and 72°C for 10 s; 95°C for 1 min; 40°C for 1 min; a melt of 72-92°C (0.01°C/s, 45 acquisitions/°C); and 40°C for 10 s. Normalized and temperature-adjusted melting curves of test samples and wild-type controls were visualized with accompanying Gene Scanning software. Since Standards (In Run) analysis mode (grouping method) was selected, the software assigned each sample to a group based on melting standard samples included in the run (wild-type replicates). Amplicons displaying abnormal melting patterns as compared to wild-type samples could be recovered from the plate, column purified and subjected to direct sequencing as described above.

## Results

### Clinical evaluation

The index patient was female, born in 1937, and had a history of allergic rhinitis, adenoid surgery, 2 vaginal childbirths, and hysterectomy due to uterine prolapse. In 2007, she consulted for constipation with some degree of urinary and fecal incontinence and was diagnosed with rectocele and rectal prolapse. One year later, a large and sessile serrated adenoma of the rectum was detected and the patient underwent transanal endoscopic microsurgery. The pathological examination of the surgical specimen revealed an infiltrating adenocarcinoma (T1) and colonoscopic follow-up was scheduled. After 17 months, she was found to have neoplastic recurrence (T3p N1) with multiple liver metastases and was considered for palliative chemotherapy with mFOLFOX6. At that time, she was slightly overweight (BMI of 29.2 kg/m^2^: weight 64 kg, height 148 cm) without other cardiovascular risk factors, such as smoking or hypertension and showed normal liver and renal function. The first cycle, administered on January 12, 2010, involved a 2-h infusion (i.v.) of oxaliplatin (85 mg/m^2^) and leucovorin (200 mg/m^2^), followed by administration of a 5-FU bolus (400 mg/m^2^ i.v.) and 48-h continuous infusion of 5-FU (2400 mg/m^2^ i.v.) using a portable pump.

On day 6 of this treatment, the patient presented to the emergency department after 3–4 days of vomiting and diarrhea, intolerance to liquids and solids, and general malaise without fever or abdominal pain, despite having taken the prescribed ondansetron. After receiving symptomatic medication consisting of pantoprazole, metoclopramide, paracetamol, and serum therapy, she remained hemodynamically stable and was admitted to the oncology service diagnosed with grade 3 mucositis. Despite a moderate initial improvement, the oral mucositis persisted and worsened to grade 4. Over the next few days, fluconazole treatment and morphine (s.c.) analgesia were given, and total parenteral nutrition was instituted. Prophylactic filgastrim and ciprofloxacin were given due to the severity of the mucositis and the presence of afebrile grade 3 neutropenia (0.58 × 10^9^/l), though the development of grade 4 thrombocytopenia required platelet transfusion. Of note, the diarrhea persisted during the entire admission period. After the appearance of fever and grade 4 neutropenia (0.02 × 10^9^/l), the antibiotic coverage was extended to piperacillin/tazobactam, but the patient developed septic shock and vasoactive drugs had to be perfused. Despite all the measures taken, the patient progressed poorly and died on January 28, 16 days after the first 5-FU dose.

### Sequence analysis of the *DPYD* gene

**Figure 1:**
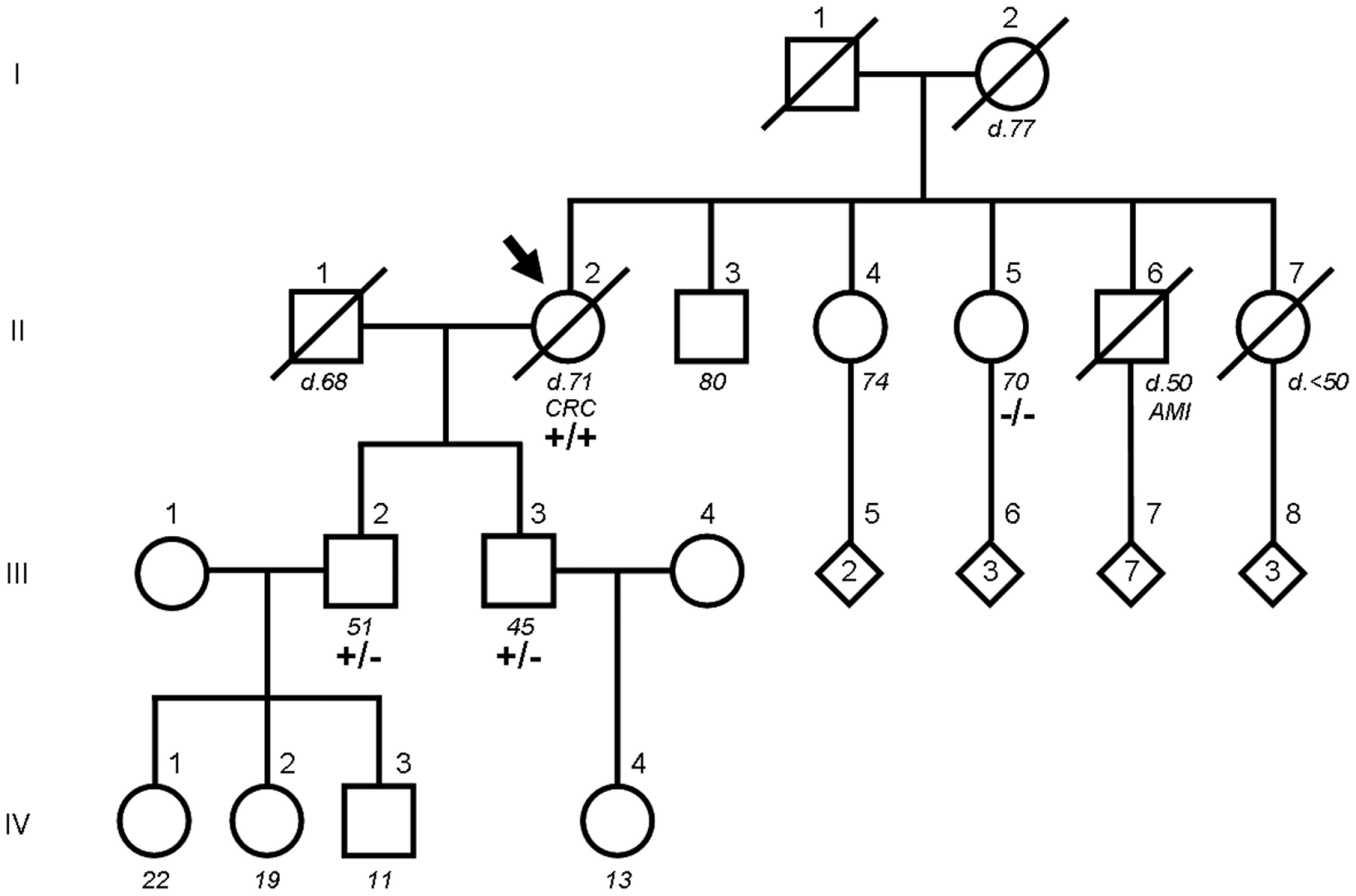
**Pedigree of the index patient and family members carrying the *DPYD* c.1905+1G>A mutation.** The index patient is indicated by an arrow. For family members who were alive, age at the time of the study is shown below in italics. For deceased members, designated by a diagonal line through the symbol, age and cause of death are annotated (CRC, colorectal cancer; AMI, acute myocardial infarction). Numbers inside a diamond are children of unspecified sex. *DPYD* genotypes are wild type (−/−), heterozygous (+/−) and homozygous (+/+) carriers of the mutation.

**Figure 2:**
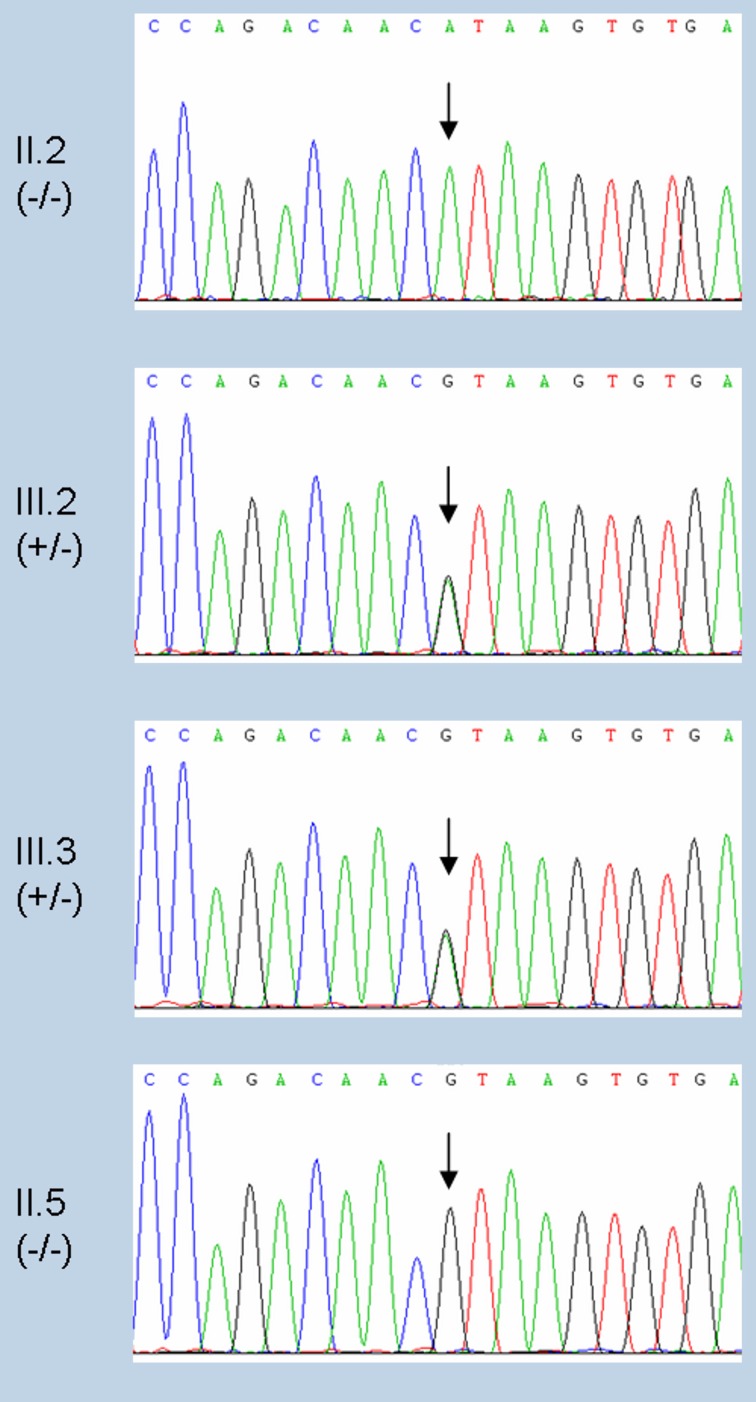
**Sequence chromatograms of the index patient and family members analyzed for the *DPYD* c.1905+1G>A mutation.** Trace sequences of *DPYD* exon 14 coding and flanking intron region including position c.1905 (indicated with an arrow). From top to bottom: c.1905+1G>A homozygous (index patient, II.2), heterozygous (sons, III.2 and III.3), and wild type (sister, II.5).

Direct sequencing of the 239-bp amplicon containing the *DPYD* exon 14 coding and flanking intron region revealed that the index patient was homozygous for the c.1905+1G>A mutation (II.2 in Figures 1, 2). Although no functional test could be performed due to unavailability of fresh blood samples, DPD activity was assumed to be completely absent according to a prior study describing the fatal outcome of a c.1905+1G>A homozygous patient with no significant residual activity of DPD in peripheral blood mononuclear cells and fibroblasts (van Kuilenburg et al., 2001).

The family study by PCR amplification followed by direct sequencing confirmed that both sons of the index patient were obligate heterozygotes (III.2 and III.3 in Figures 1, 2) and the only sister analyzed was wild type (II.5 in Figures 1, 2). Of note, none of the mutation carriers of this family presented symptoms of familial pyridinemia and DPD deficiency was not discovered until the administration of 5-FU. To date, none of the other siblings of the index patient are available for testing. Nevertheless, as the members studied represent the three genotypes, their DNA samples were used to evaluate the validity of the HRM assay.

### HRM analysis of the *DPYD* c.1905+1G>A mutation

**Figure 3:**
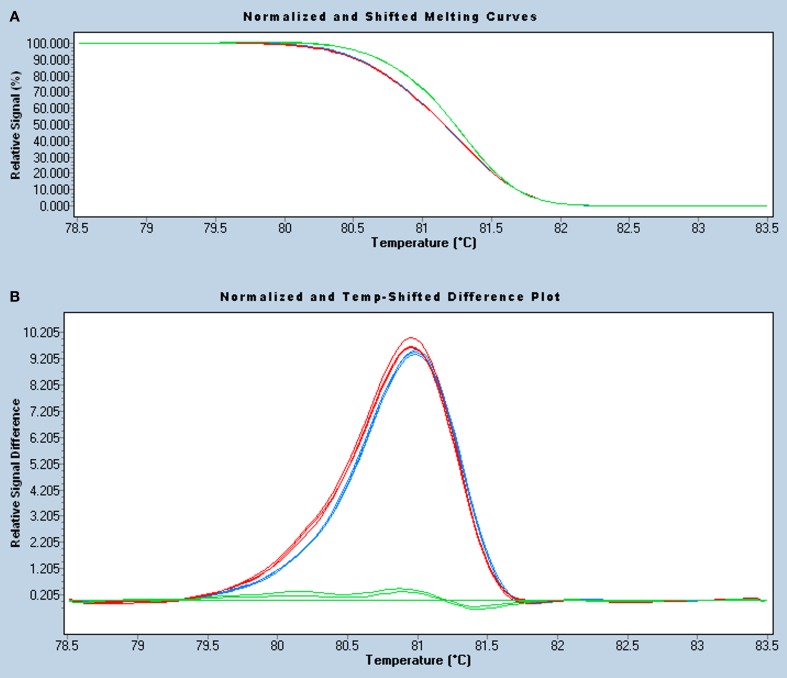
**HRM analysis of the *DPYD* c.1905+1G>A mutation in a 239-bp amplicon.** Normalized and temperature-shift melting curves **(A)** and differential plot **(B)** of mutant homozygous (II.2, blue), heterozygous (III.2, red), and wild-type (II.5, green) samples, assayed in triplicate. As all samples were spiked with wild-type DNA, homozygous and heterozygous mutants show similar left-shifted melting curves and can be easily identified, especially in the differential plot.

The HRM assay developed was tested using triplicates of the above mentioned samples, adjusted to the same concentration and spiked with wild-type DNA. The homozygous mutant sample showed the lowest DNA concentration (16 ng/μl) as a result of the severe neutropenia, so the other samples were diluted accordingly. Homozygous and heterozygous carriers of the *DPYD* c.1905+1G>A mutation were successfully identified by HRM analysis, either using the adjusted melting curves (Figure 3A) or the differential plot (Figure 3B). The adjusted melting curves show a single melting domain, consistent with Stitchprofiles.uio.no predictions, but, since the melting curves of mutation carriers are a composite of both heteroduplex and homoduplex components, they dissociate more readily and shift left to a lower temperature. The difference plot calculation assigned the samples in two groups using sensitivity values from 0.2 (higher values denote high stringency and produce more groups), so the mutant samples (either homozygous or heterozygous) were distinguished from the wild-type ones (melting standards). For good HRM analysis, amplification curves were checked to produce a crossing point <30 and to reach a similar plateau height and, if replicates showed different melting patterns, the assay was repeated for that sample. Finally, the touchdown PCR and melting conditions of this *DPYD* HRM assay were suitable for analysis of *KRAS*, *BRAF*, and *EGFR* somatic hotspot mutations in tumor samples (not shown), thus enabling simultaneous analysis of relevant mutations for targeted cancer therapy.

## Discussion

To date, screening for the presence of DPD deficiency prior to 5-FU chemotherapy is not yet established in the daily care of cancer patients, despite the numerous studies worldwide reporting life-threatening toxicity cases. Therefore, while not specified in professional guidelines, there is general consensus that given the large number of patients treated with 5-FU and the human and economic cost of grade 3–4 toxic side effects, DPD deficiency should be tested for prior to initiation of therapy.

Several methods have been developed to assess DPD activity, such as direct assays in peripheral blood mononuclear cells, indirect evaluations by monitoring DPD substrates or metabolites (e.g., uracil/dihydrouracil plasma ratio, uracil breath test), administration of a 5-FU test dose, and measurement of DPD expression through mRNA or protein levels (Mercier and Ciccolini, 2006; Eidens et al., 2009). However, functional tests usually require special equipment and are too costly and laborious for routine implementation in clinical practice. In contrast, genotyping methods are available in most laboratories but offer an incomplete pharmacogenetic diagnosis because of the limited number of genetic variants tested and the lack of a straightforward genotype-phenotype correlation. Combining the two approaches may provide the most complete assessment of toxicity risk, although no guidelines currently exist specifying a particular testing method.

Regarding genotyping, comprehensive genetic screenings including the variation in noncoding regions result in a higher relative importance of *DPYD* variants to explain 5-FU toxicities. Current data suggest that combining information from multiple variants in this gene can identify over 20% of patients experiencing severe 5-FU toxicity (Amstutz et al., 2011). On the other hand, heterozygous carriers of deleterious variants can show normal DPD activity and only about 50% of carriers develop severe 5-FU toxicity, which suggests an allelic regulation through an increased expression of the wild-type allele or compensation by another variant that confers above-average DPD activity (Amstutz et al., 2011). Whatever the case, extensive analyses of *DPYD* could address this issue. Moreover, genotype testing could be expanded to genetic variants in genes that may play a role in 5-FU breakdown, such as thymidilate synthase (*TYMS*) and methylenetetrahydrofolate reductase (*MTHFR*), which could modulate the impact of *DPYD* risk alleles on the overall risk of toxicity.

High-throughput sequencing technologies promise to substantially simplify this task in the future, as full sequencing of *DPYD* and other genes of potential importance for 5-FU toxicity will be achieved at reasonable costs. Further comprehensive genetic screenings in combination with phenotypic characterization of *DPYD* genotypes could help to identify the factors underlying the occurrence of normal DPD activity in carriers of risk alleles and to discern the relative contribution of individual *DPYD* variants. Meanwhile, methodologies based on genetic testing for clinically relevant variants offer the simplest way to identify patients at the highest risk of potentially life-threatening adverse drug events.

In this context, we describe an approach to detect the c.1905+1G>A mutation of *DPYD* based on HRM technology, which shows great potential for scanning germline and somatic mutations (Taylor, 2009). A HRM assay previously designed by our group (Borràs et al., 2011) had already successfully identified hotspot mutations of *KRAS*, *BRAF*, and *EGFR* with a high analytical sensitivity. Moreover, the use of a touchdown PCR and a wide melting interval allowed the simultaneous analysis of all amplicons in a single plate, saving time and cost. Although this assay was developed for FFPE tumor sections, it is also suitable for blood samples, and both DNA sources can be combined in one experiment to detect somatic and germline mutations. As DNA isolation from blood samples usually gives higher yields and better quality, lower amounts of template could be used, but the amount of starting DNA has to be standardized as much as possible to minimize reaction-to-reaction variability.

Since this test has to be validated before it is used in routine, we plan to conduct a pilot study in our institution to genotype the *DPYD* c.1905+1G>A mutation in cancer patients and to assess its importance in 5-FU toxicity. Considering that 5-FU is widely prescribed for the treatment of solid carcinomas, like those of the gastrointestinal tract, pretreatment *DPYD* genotyping could be performed together with the detection of *KRAS* and *BRAF* somatic mutations in patients with colorectal cancer to predict the response to anti-*EGFR* monoclonal antibodies, recommended by regulatory authorities (van Krieken et al., 2008; Allegra et al., 2009; NCCN Colon Cancer Guidelines, 2011).

Just as better mutation detection methods are required for stratification of patients to receive molecularly targeted treatment, tests are needed for the cost-effective screening of genes associated with drug metabolism and response. Understanding pharmacogenetic associations is especially important in cancer chemotherapy, as many chemotherapeutic agents, such as 5-FU, have a very narrow therapeutic index. Among the various techniques available to detect DPD deficiency at genotype level, including many marketed tests, HRM analysis provides a rapid, sensitive, and inexpensive method that can be easily implemented in a diagnostic setting.

HRM has been applied to mutation scanning of the cytidine deaminase gene (*CDA*), involved in the catabolism of nucleoside analogs, genetic variations of which might explain the therapeutic and toxic response to gemcitabine (Evrard et al., 2007a,b). Specifically, the LightCycler® 480 platform was used to investigate variations in long PCR fragments and to genotype SNPs or mutations in short amplicons, and HRM efficiently identified single base heterozygous changes in PCR products up to 622 bp. However, differentiation of homozygous variants depended on amplicon length and GC content, so the use of modified DNA is suggested. Spikes of wild-type DNA added to all samples and comparison to unspiked reactions has been shown by others to provide a valuable approach to addressing this point. Furthermore, the authors compare three methods for routine detection of c.1905+1G>A mutation in the *DPYD* and consider HRM to be a powerful tool for genotyping known SNPs or mutations in routine clinical practice (Evrard et al., 2007a,b). However, just like any screening test, HRM-identified positive samples have to be subsequently sequenced to identify the specific nucleotide alteration, which may be present in one or both alleles, and to avoid misdiagnosis due to an abnormal curve generated by a neutral variant.

An important limitation of our study is that screening for the c.1905+1G>A mutation alone may have limited effectiveness in identifying patients at risk of lethal 5-FU toxicity and could result in false-negative results for patients with rare *DPYD* variants or who might experience severe toxicity as a result of other causes. Prospective analysis of the c.1905+1G>A mutation in large numbers of toxicity cases and controls from our population is needed for a reliable estimation of the importance of this variant for the prediction of 5-FU toxicity in cancer patients and to determine the cost-effectiveness of a genetic strategy for DPD screening.

In this study, we describe the case of a woman with an unremarkable medical history before the diagnosis of colorectal cancer followed by surgery and 5-FU-based chemotherapy, with subsequent unexpected gastrointestinal and hematologic toxicity leading to death. As in other reports, most patients have no symptoms of DPD deficiency and are unaware of their condition prior to 5-FU treatment and the subsequent development of adverse side effects. In contrast, both sons of the index patient are known to be heterozygous carriers of the c.1905+1G>A mutation but the clinical implications of partial DPD deficiency are unpredictable since not everybody who carries the risk allele may actually suffer severe 5-FU side effects. In these cases, determination of the 5-FU pharmacokinetics could aid individualized therapy since the application of dose-tailored strategies based on pharmacokinetic monitoring improved the therapeutic index of 5-FU treatment, and it could be used in conjunction with genotyping to reduce toxicity and achieve maximum benefit (Saif et al., 2009; Yang et al., 2011).

As a standard practice, many authors have suggested that patients with decreased DPD activity should be monitored closely, considered for a reduced 5-FU dose, or chosen for an alternative therapy (Raida et al., 2001; Lazar and Jetter, 2008; Ciccolini et al., 2010). More recently, the Pharmacogenomics Working Group of the Royal Dutch Association for the Advancement of Pharmacy established clinical guidelines for 5-FU therapy according to *DPYD* genotype, available at the Pharmacogenomics Knowledge Base (www.pharmgkb.org). For patients carrying two inactive or decreased activity alleles, they recommend selecting an alternative drug, whereas for patients with one active and one inactive or decreased activity allele, a 50% dose reduction or selection of another drug is recommended.

With the aim of contributing toward the implementation of a pre-screening program for DPD deficiency and helping to improve the safety of 5-FU-based chemotherapy, we have developed a HRM assay for the screening of *DPYD* c.1905+1G>A mutation. This method provides a simple, robust, and inexpensive solution that can be easily implemented in diagnostic settings for pre-therapy testing. Therefore, after proper validation for routine use, we plan to include the present test in a panel of other tests for somatic cancer mutations with implication on the selection of therapy.

### Conflict of interest statement

The authors declare that the research was conducted in the absence of any commercial or financial relationships that could be construed as a potential conflict of interest.
